# Oxidation of the carcinogenic non-aminoazo dye 1-phenylazo-2-hydroxy-naphthalene (Sudan I) by cytochromes P450 and peroxidases: a comparative study

**DOI:** 10.2478/v10102-009-0017-z

**Published:** 2009-09-28

**Authors:** Marie Stiborová, Václav Martínek, Marcela Semanská, Petr Hodek, Martin Dračínský, Josef Cvačka, Heinz H. Schmeiser, Eva Frei

**Affiliations:** 1 Department of Biochemistry, Faculty of Science, Charles University, Albertov 2030, 128 40 Prague 2, Czech Republic; 2 Institute of Organic Chemistry and Biochemistry, v.v.i., Academy of Sciences of the Czech Republic, Flemingovo n. 6, 166 10 Prague 6, Czech Republic; 3 German Cancer Research Center, Im Neuenheimer Feld 280, 69120 Heidelberg, Germany

**Keywords:** carcinogenic azo dye, Sudan I, cytochrome P450, peroxidase, oxidative activation

## Abstract

Sudan I [1-(phenylazo)-2-hydroxynaphthalene, C.I. Solvent Yellow 14, CAS No: 842-07-9] is used as the compound employed in chemical industry and to color materials such as hydrocarbon solvents, oils, fats, waxes, plastics, printing inks, shoe and floor polishes and gasoline. Such a wide used could result in a considerable human exposure. Sudan I is known to cause developments of tumors in the liver or urinary bladder in rats, mice, and rabbits, and is considered a possible weak human carcinogen and mutagen. This carcinogen is also a potent contact allergen and sensitizer. Here, we compare the data concerning the Sudan I oxidative metabolism catalyzed by cytochrome P450 (CYP) and peroxidase enzymes, which has been investigated in our laboratory during the last two decades. These two types of enzymes are responsible both for Sudan I detoxication and activation. Among the Sudan I metabolites, C-hydroxylated derivatives and a dimer of Sudan I are suggested to be the detoxication metabolites formed by CYPs and peroxidases, respectively. Metabolic activation of Sudan I by both types of enzymes leads to formation of reactive species (the benzenediazonium ion by CYP and Sudan I radicals by peroxidase) that bind to DNA and RNA, generating covalent adducts *in vitro* and *in vivo*. Whereas the structure of the major adduct formed by the benzenediazonium ion in DNA has already been identified to be the 8-(phenylazo)guanine adduct, the structures of adducts formed by peroxidase, have not been characterized as yet. Biological significance of the DNA adducts of Sudan I activated with CYP and peroxidase enzymes and further aims of investigations in this field are discussed in this study.

## Introduction

Azo dyes are a large group of chemically related compounds widely used for industrial purposes and as colorants of materials of daily use. Organic azo colorants are grouped into several types, depending on similarity in the chemical structure. One of such types is a group of azo compounds that are structurally similar to Sudan I [1-(phenylazo)-2-hydroxynaphthalene, C.I. Solvent Yellow 14, CAS No: 842-07-9, Figure 1)].

**Figure 1:**
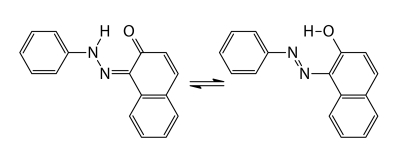
Sudan I (keto and enol forms).

## Toxicity, mutagenicity and carcinogenicity of Sudan I

Sudan I was used as a food coloring in several countries (IARC, 1975), but it has been recommended as unsafe, because of its potential toxicity and carcinogenicity. This dye was found to cause developments of tumors in the liver or urinary bladder in rats, mice, and rabbits, and is considered a possible weak human carcinogen and mutagen (NCI, 1982; Garner *et al*., 1984; Westmoreland and Gatehouse, 1991; Moller and Wallin, 2000; An *et al*., 2007; Zhang *et al*., 2008a). Besides its carcinogenicity, Sudan I is a potent contact allergen and sensitizer, eliciting pigmented contact dermatitis in human (Kozuka *et al*., 1980). Nevertheless, it is used to color materials such as hydrocarbon solvents, oils, fats, waxes, plastics, printing inks, shoe and floor polishes and gasoline (IARC, 1975; Moller and Wallin, 2000). Moreover, Sudan I is an important compound, not because it is still used to color these materials, but because it is the simplest in a series of dyes and pigments including Sudan III and Sudan IV that are used in great quantities and occur everywhere in red and orange colored consumer products, foods and printed matter. Besides Sudan I, Sudan III and Sudan IV have also been found to be weak carcinogens, being classified as category 3 carcinogens by International Agency for Research on Cancer (IARC, 1975; Moller and Wallin, 2000; *http://msds.chem.ox.ac.uk/SU/sudan_I.html*). Even though the amounts of these azo dyes used in industry and to color the above materials have not yet been exactly evaluated, their use causes long-term harm in the environment and could result in a considerable human exposure (IARC, 1975; Moller and Wallin, 2000; *http://msds.chem.ox.ac.uk/SU/sudan_I.html*). Recently, an increased attention has been paid to this dye, because it has been found as a contaminant of several European foodstuffs, being detected in chilli powder and in chilli-containing food products such as in Pixian douban, Golden Mark guilin chilli sauce, Golden Mark satay sauce, Italian pasta, chilli-snack and vegetable sauce (Mazzetti *et al*., 2004; Liu *et al*., 2007; Uematsu *et al*., 2007; Wang *et al*., 2007). More recently, levels of these dyes were evaluated; analysis of a few market samples of turmeric, chilli, and curry powders showed the presence of Sudan I (4.8–12.1 mg/g), Sudan IV (0.9–2.0 mg/g) and metanil yellow (1.5–4.6 mg/g) in loose turmeric and chilli samples (Xu *et al*., 2007; Dixit *et al*., 2008; Wang *et al*., 2009). In addition, there is evidence that ingestion of food products contaminated with Sudan I could lead to exposure in the human gastrointestinal tract to metabolites generated by the intestinal bacteria (Xu *et al*., 2007). Therefore, the use of Sudan I as an additive in food products has been prohibited in the European Union and many other countries (Federal Institute for Risk Assessment, 2003). Nevertheless, the question whether the recent detection of Sudan I and other Sudan I-derived dyes in various food commodities and additional materials is actually serious problems requires further toxicological evaluations by regulatory agencies. Such evaluations should determine the real impact of this Sudan I dye on human health (Federal Institute for Risk Assessment, 2003; Mazzetti *et al*., 2004; Liu *et al*., 2007; Uematsu *et al*., 2007; Wang *et al*., 2007; Xu *et al*., 2007; Dixit *et al*., 2008; Wang *et al*., 2009).

Sudan I gives positive results in *Salmonella typhimurium* mutagenicity tests with S-9 activation (Cameron *et al*., 1987; Zeiger *et al*., 1988) and is mutagenic to mouse lymphoma L5178Y TK^+/−^ cells *in vitro*, with S-9 activation (Zeiger *et al*., 1988). It is a clastogenic compound, inducing micronuclei in the bone marrow of rats (Westmoreland and Gatehouse, 1991). There is also evidence that this compound exhibits genotoxic effects, after its metabolic activation by hepatic cytochrome P450 (CYP) and peroxidase enzymes *in vitro*, and in the rat liver and urinary bladder *in vivo* (Stiborová *et al*., 1988b; c; 1990a; b; 1992; 1993; 1995a; b; 1999a; b; 2002; 2006; Dixit *et al*., 2008; Zhang *et al*., 2008b,), and in a human hepatoma cell line, HepG2 (An *et al*., 2007; Zhang *et al*., 2008b).

## Activation and detoxication metabolism of Sudan I catalyzed by cytochromes P450

**Figure 2:**
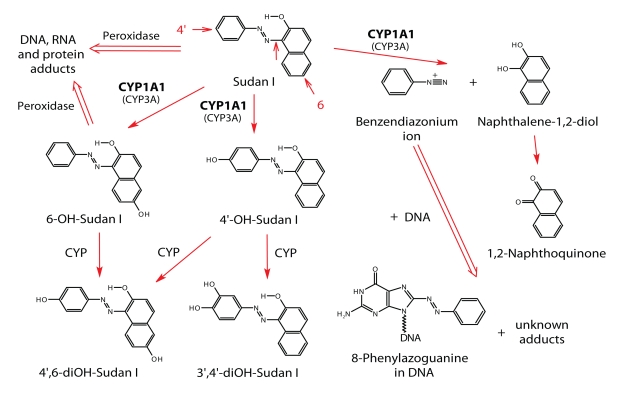
Scheme of Sudan I metabolism.

**Figure 3:**
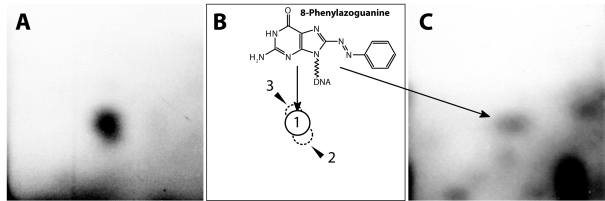
Autoradiographs of PEI-cellulose thin layer chromatography (TLC) maps of ^32^P-labeled digests of calf thymus DNA reacted with Sudan I, NADPH and human recombinant CYP1A1 in Supersomes (**A**) and of liver DNA of rats treated with Sudan I (**C**). (**B**) Schematic figure of adducts with assigned numbers and the structure of adduct 1 (closed circle). Analysis was performed by the nuclease P1 version of the assay. Chromatographic conditions are described (Stiborová *et al*., 1995; 2006). Autoradiography was at -80°C for 6 (A) and 3 h (C). Origins are located in the bottom left corners (Stiborová *et al*., 2006).

While the metabolism of Sudan I is not understood in humans, its metabolism has been characterized in rabbits (Childs and Clayson, 1966), where it is metabolized primarily in the liver by oxidative or reductive reactions (Childs and Clayson, 1966). Azo-reduction of Sudan I seems to be responsible mainly for its detoxification, producing aniline and 1-amino-2-naphthol (Moller and Wallin, 2000). C-Hydroxylated metabolites 1-(4-hydroxyphenylazo)-2-naphthol (4′-OH-Sudan I), 1-(phenylazo)-naphthalene-2,6-diol (6-OH-Sudan I) and 1-(4-phenylazo)-naphthalene-2,6-diol (4′,6-diOH-Sudan I) were found to be the major products of Sudan I oxidation *in vivo* and excreted in urine (Childs and Clayson, 1966; IARC, 1975), and also of its oxidation by rat hepatic microsomes *in vitro* (Stiborová *et al*., 1988a) (Figure 2). Glucuronides of the same three compounds have been detected in bile and urine of rabbits fed Sudan I (Childs and Clayson, 1966). Another C-hydroxylated metabolite, 1-(3,4-dihydroxyphenylazo)-2-naphthol (3′,4′-diOH-Sudan I), was found to be formed as a minor product by Sudan I oxidation with rat hepatic microsomes *in vitro* (Stiborová *et al*., 1994) (Figure 2). Besides the C-hydroxylated metabolites, which are considered detoxication products, the benzenediazonium ion (BDI), formed by microsome-catalyzed enzymatic splitting of the azo group of Sudan I, was found to react with DNA *in vitro* (Stiborová *et al*., 1988a; b; 1995b). The major DNA adduct formed in this reaction has been characterized and identified as the 8-(phenylazo)guanine adduct (Stiborová *et al*., 1995b) (Figure 2). This adduct was also found in liver DNA of rats exposed to Sudan I (Stiborová *et al*., 2006) (Figure 3). Oxidation of Sudan I with formation of the same C-hydroxylated metabolites and Sudan I-derived DNA adducts was also demonstrated with human CYP enzymes (Stiborová *et al*., 2002 2005). CYP1A1 is the major enzyme oxidizing Sudan I in human tissues rich in this enzyme, while CYP3A4 is also active in human liver (Stiborová *et al*., 2002 2005) (Figure 2). Interestingly, even though levels of CYP1A1 expression in human livers are low, <0.7% of total hepatic CYP, the CYP1A1 contribution to oxidation of carcinogenic Sudan I in the set of human liver microsomes of Caucasian donors tested in our former study ranges from 12 to 30% (Stiborová *et al*., 2005). Moreover, the CYP1A1 enzyme is strongly induced by Sudan I itself in rats and human cells in culture, due to activating the cytosolic aryl hydrocarbon receptor (Lubet *et al*., 1983). Hence, long-term occupational exposure of humans to Sudan I might be an important risk factor for individuals, improving Sudan I metabolism and binding to DNA, thereby increasing its toxicological relevance.

## Activation and detoxication metabolism of Sudan I by peroxidases

While microsomal CYPs were found to be responsible for the activation of Sudan I in human or animal liver (Stiborová *et al*., 1988b; 1995b; 2002; 2005; Martínek and Stiborová 2002), they play a marginal role in the *in vivo* metabolic activation of Sudan I in the urinary bladder, because this organ has little or no detectable CYP enzymes. But relatively high levels of peroxidases are expressed in this tissue (Wise *et al*., 1984). We have found that in addition to microsomal CYP enzymes, Sudan I and its C-hydroxylated metabolites are also oxidized by peroxidases such as a model plant peroxidase from horseradish as well as the mammalian enzyme, prostaglandin H synthase (cyclooxygenase), as a consequence DNA, RNA and protein adducts are formed (Stiborová *et al*., 1988c; 1990a;b; 1991; 1992; 1993; 1995a; 1999b) (Figure 2). In bladder, therefore, peroxidase-catalyzed activation of Sudan I has been suggested, similar to other carcinogens such as carcinogenic aromatic amines (Wise *et al*., 1984; Yamazoe *et al*., 1985; Eling *et al*., 1990; Chen et al., 1996). We have suggested a CYP- or peroxidase-mediated activation of Sudan I or a combination of both mechanisms as an explanation for the organ specificity of this carcinogen for liver and urinary bladder in animals (Stiborová *et al*., 1988b; 1990a;b; 1992; 1995b; 2002; 2005; Martínek and Stiborová, 2002). Indeed, the 8-(phenylazo)guanine DNA adduct generated from the BDI, the product of CYP activation, was found in the liver of rats treated with Sudan I (Stiborová *et al*., 2006) (Figure 3), whereas the physico-chemical properties of DNA adducts found in the urinary bladder are identical to those formed by the peroxidase-mediated Sudan I activation that contain the whole molecule of Sudan I (Stiborová *et al*., 1999a).

**Figure 4:**
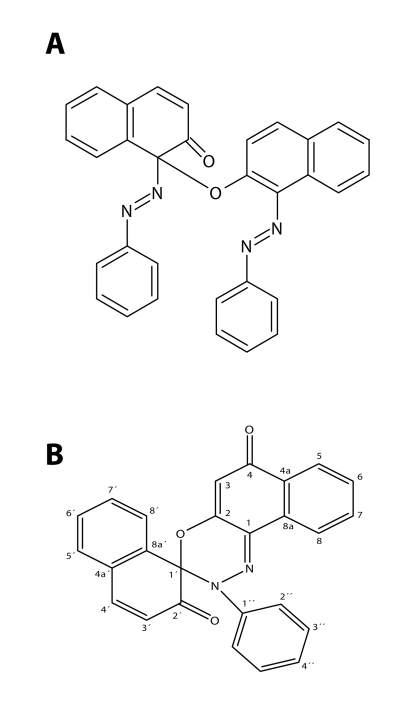
Sudan I metabolites generated during its peroxidase mediated oxidation. (**A**) Sudan I dimer, (**B**) spiro-bezoxadiazine derivative of Sudan (Semanská *et al*., 2008).

Unfortunately, neither the structures of DNA- or RNA-adduct(s), nor those of the ultimate carcinogen(s) formed by peroxidase from Sudan I are known as yet. This knowledge is, however, crucial for the comparative study with the *in vivo* products (Stiborová *et al*., 1999a, 2006). In our former studies with peroxidase activation of Sudan I, we have identified BDI and C-hydroxy derivatives of Sudan I [6-OH-Sudan I and 4′,6-di(OH)-Sudan I] as only minor metabolites, while products of a suggested polymerization of the primarily formed Sudan I radicals were more abundant (Stiborová *et al*., 1988c; 1991; 1996). In the meantime, we have confirmed this for the two major metabolites (Semanská *et al*., 2008), one is a Sudan I dimer (Figure 4A) generated by a Sudan I one electron oxidation, while the other is the product of secondary, enzyme independent reactions of this Sudan I dimer, spiro-bezoxadiazine derivative of Sudan I (Semanská *et al*., 2008) (Figure 4B). Plant horseradish peroxidase (HRP) was found in these studies to be an acceptable model for Sudan I oxidation by mammalian enzymes such as non-specific urinary bladder peroxidases and/or cyclooxygenases (Stiborová *et al*., 1990a;b; 1996; 2000; 2002; 2006). Even though mammalian and plant peroxidases are structurally different proteins, the oxidation mechanisms are similar due to analogous arrangements of their active sites (Wise *et al*., 1984; Yamazoe *et al*., 1985; Eling *et al*., 1990; Stiborová *et al*., 1991, 2000; Chen et al., 1996).

It should be noted that the Sudan I metabolites formed by peroxidase (Figure 3) are much less likely to be formed physiologically than in the *in vitro* system (Semanská *et al*., 2008), because many nucleophilic molecules are present in cells to scavenge the Sudan I reactive species. Indeed, during Sudan I oxidation by peroxidase in the presence of nuclephiles such as DNA, tRNA polydeoxynucleotides, polynucleotides and or proteins, the formation of adducts runs parallel to a decrease in generation of Sudan I metabolites (Stiborová *et al*., 1990a;b; 1991; 1999b; and Stiborova *et al*., unpublished data). Hence, formation of adducts of Sudan I reactive species with these nucleophilic compounds seems to be the preferred reaction under physiological conditions.

**Figure 5:**
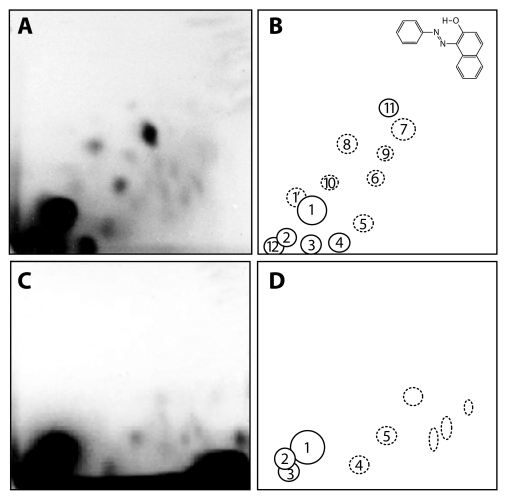
Autoradiographs of PEI-cellulose TLC maps of P-labeled digests of the following: (**A**) rat liver tRNA treated with Sudan I, peroxidase and hydrogen peroxide, (**C**) polyguanosine treated with Sudan I, peroxidase and hydrogen peroxide. (**B**) and (**D**) Schematic figures of adducts formed in tRNA (**B**) and polyguanosine (**D**) with assigned numbers. Analysis was performed by the nuclease P1 enhanced version of the assay. Chromatographic conditions are described (Stiborová *et al*., 1990a; b; 2002). Autoradiography was at 25 °C for 25 min (**A**) and for 5 min (**C**). Origins are located at the bottom left corners (D3 from bottom to top and D4 from left to right).

Using the ^32^P-postlabeling assay (Randerath *et al*., 1981; Gupta 1985) we have already analyzed Sudan I-DNA adducts formed by peroxidases (HRP, cyclooxygenase) (Stiborová *et al*., 1990a;b; 1992; 1999b). Deoxyguanosine was the major target for Sudan I-DNA binding, followed by deoxyadenosine (Stiborová *et al*., 1992). Likewise, guanosine was found to be the major target for peroxidase-activated Sudan I binding in RNA (Stiborová *et al*., 1995a). Autoradiographs of thin layer chromatography (TLC) maps of ^32^P-labelled digests of tRNA and polyguanosine modified by Sudan I activated with peroxidase, shown in Figure 5, also indicate that guanosine is the target for binding of Sudan I activated with peroxidase in tRNA.

It has been postulated by Eling and coworkers (Eling *et al*., 1990) that characterization of peroxidase-mediated adducts derived from carcinogens is exceptionally difficult due to problems in preparing the DNA (or RNA) adducts in sufficient quantities and purity for structural analysis. Indeed, our earlier studies with peroxidase activation of Sudan I in the presence of DNA, tRNA, (deoxy)guanosine 3′-monophosphate or (deoxy)guanosine 5′-monophosphate did not yield enough adducts for their structural characterization (Stiborová *et al*., 1992; 1995a).

## Conclusion

Based on the above findings, the study which will lead to prepare and isolate deoxyguanosine- and guanosine adducts formed by the peroxidase-activated Sudan I in sufficient amounts and purity is essential for their structural characterization. Therefore, such a study is under way in our laboratory. It will contribute to establish the molecular mechanisms explaining the formation of two Sudan I metabolites formed by peroxidase (Figure 3) and contribute to determine structures of adducts that were found to be formed in DNA/RNA *in vitro* (Stiborová *et al*., 1990a;b; 1992; 1995a;b; 1999b) and *in vivo* (Stiborová *et al*., 1999a; 2006).
